# Impact of postoperative delirium on long-term neurologic and neuropsychiatric outcome after cardiac surgery or percutaneous valve replacement–a prospective observational study

**DOI:** 10.3389/fcvm.2025.1635225

**Published:** 2025-11-24

**Authors:** Maike Schmidt, Lukas Hinken, Johannes Teller, Maria Magdalena Gabriel, Svea-Dorothee Schimmelpfennig, Ralf Lichtinghagen, Christine Fegbeutel, Andreas Schäfer, Karin Weissenborn, Carolin Jung, Hans Worthmann

**Affiliations:** 1 Department of Neurology, Hannover Medical School, Hanover, Germany; 2 Institute of Diagnostic and Interventional Radiology, University Hospital of Cologne, Cologne, Germany; 3 Department of Anesthesiology, Hannover Medical School, Hanover, Germany; 4 Department of Clinical Chemistry, Hannover Medical School, Hanover, Germany; 5 Department of Cardiac, Thoracic, Transplantation and Vascular Surgery, Hannover Medical School, Hanover, Germany; 6 Department of Cardiovascular Surgery, Hannover Medical School, Hanover, Germany; 7 Department of Cardiology and Angiology, Hannover Medical School, Hanover, Germany

**Keywords:** postoperative delirium, cardiac surgery, percutaneous valve replacement, neurologic and neuropsychiatric outcome, cognitive function, depression

## Abstract

**Background:**

Patients undergoing cardiac surgery or percutaneous valve replacement may experience long-term neurologic and neuropsychiatric complications. The impact of postoperative delirium (POD) on these long-term complications remains controversial. We intended to illustrate the neurological and neuropsychiatric outcome associated with the occurrence of POD in patients undergoing elective cardiac surgery or percutaneous valve replacement.

**Methods:**

We included 179 patients who underwent elective cardiac surgery or percutaneous valve replacement. Patients were evaluated postoperatively for delirium status. Neurological (score A) and neuropsychiatric (score B) outcomes were assessed using a structured examination protocol and interview at 1 year postoperatively and combined into a composite neurological and neuropsychiatric score (score A + B). Cognitive function was examined using the Montreal Cognitive Assessment (MoCA). Depression, fatigue and quality of life were assessed using the Beck's Depression Inventory (BDI), the Fatigue Impact Scale (FIS) and the Short-Form Health Survey (SF-12). Clinical outcome was assessed using the Barthel-Index (BI) and Frailty Index (FI). All data were collected prospectively.

**Results:**

One year after cardiac surgery or percutaneous valve replacement, a high number of patients suffered from neurological and neuropsychiatric symptoms with depressive symptoms (*n* = 36, 20.1%) and symptoms of fatigue (*n* = 72, 40.2%). Multivariable regression analysis showed that POD was associated with higher values on the A + B composite score, indicating worse neurological and neuropsychiatric outcome (POD status: b:1.172; 95%-CI, 0.070–2.273, *p* = 0.037; age: b:0.134; 95%-CI, 0.086–0.182, *p* < 0.001; NYHA classification at 1 year: b:1.998; 95%-CI,1.169–2.828, *p* < 0.001; rehospitalization b:1.786; 95%-CI, 0.640–2.932, *p* = 0.002). Patients with POD had lower postoperative MoCA scores (*p* = 0.001) and lower scores on both the SF12 Physical (*p* = 0.022) and the SF12 Mental Component Summary (*p* = 0.048). POD was not associated with depressive symptoms (*p* = 0.855), fatigue (*p* = 0.122) or rehospitalization (*p* = 0.379).

**Conclusions:**

POD in patients undergoing cardiac surgery or percutaneous valve replacement was independently associated with worse long-term neurological and neuropsychiatric outcome. POD may be a relevant prognostic marker indicating the need for specific follow-up services, whereas other clinical parameters were not predictive of outcome.

## 1 Introduction

In patients with cardiac surgery or percutaneous valve replacement postoperative delirium (POD) is a common complication and is associated with adverse clinical outcome, as evidenced by prolonged hospital stay (1), and higher rates of morbidity and mortality (2). Studies following cardiac surgery report that cognitive function and health-related quality of life (HRQoL) are impaired in patients with POD (3, 4). A systematic review of 14,824 patients who underwent cardiac surgery demonstrated that POD (*n* = 1,130) was additionally associated with more hospital readmissions and an overall decline in functional status (5). Mechanistically, prolonged stress and inflammatory processes (6), disruption of circadian rhythms (7), nightmares and memories of the patients’ intensive care unit (ICU) stay (8) are thought to contribute to the development of neuropsychiatric disorders not only in the short term but also in the long term (9). However, in most of the studies performed, face-to-face assessments of long-term neurologic and neuropsychiatric outcome as well as prospectively collected preoperative baseline data are lacking.

The primary aim of this study was to prospectively illustrate long-term neurological and neuropsychiatric outcomes after cardiac surgery and percutaneous valve replacement and to examine the association of POD with symptoms of depression and fatigue, cognitive function, HRQoL, hospital readmission, and overall function.

## 2 Methods

### 2.1 Study population

Between July 2018 and March 2019, *n* = 320 patients undergoing cardiac surgery or percutaneous valve replacement were screened for POD at a university hospital as described in a previous study (10). Of these patients 179 were prospectively included into this study for a one-year follow-up visit (1yFU) between July 2019 and March 2020. 141 patients could not be included in the 1yFU due to refusal to participate (*n* = 63), death (*n* = 16), incomplete data (*n* = 5), inability to attend follow-up due to poor general health (*n* = 10) or current hospitalization (*n* = 7) during the study period, unavailability for contact (*n* = 7), long distance from home (*n* = 7), and early termination of the study due to the COVID-19 pandemic (*n* = 26).

The study was approved by the Ethics Committee at Hannover Medical School (local ethics committee approval No. 7876) and was conducted in accordance with the Declaration of Helsinki. Written informed consent was obtained from all study participants.

### 2.2 Postoperative delirium assessment and baseline data

At baseline and at 1-year follow-up medical history and clinical characteristics such as age, sex, NYHA classification and cardiovascular risk factors were assessed. In addition, perioperative clinical data (type, duration of surgery or number of erythrocyte concentrates received) were collected. Screening for POD was performed twice daily on the first, second and third day after cardiac surgery or percutaneous valve replacement (10). For the detection of POD either the Confusion Assessment Method (3D-CAM) or the Confusion Assessment Method Intensive Care Unit (CAM-ICU) was used, depending on the patient's level of agitation and alertness. If the Richmond Agitation-Sedation Scale (RASS)-Score was between −1 and +1, the 3D-CAM was assessed; otherwise, CAM-ICU was used. The CAM algorithm includes four features: (1) acute onset and fluctuating course, (2) inattention, (3) disorganized thinking, and (4) altered level of consciousness. The presence of both the first and the second feature and either the third or the fourth feature defines a positive screening test for POD (11).

#### 2.2.1 Clinical evaluation

1yFU took place at Hannover Medical School or at the participants’ homes. Neurological and neuropsychiatric outcome was evaluated for each patient using a preexisting protocol including a structured neurologic examination and a neuropsychiatric assessment. Score A (range: 0–88) covers neurological complaints, whereas score B (range: 0–16) covers neuropsychiatric symptoms. Score A + B includes both, neurological and neuropsychiatric symptoms (Supplementary Table S2). This score is a tool for neurological and neuropsychiatric examinations developed by our research group to comprehensively cover a wide range of possible symptoms. In order to detect and quantify the symptoms, the individual findings are aggregated, resulting in an overall assessment of neurological and neuropsychiatric impairment. It comprises 10 symptom domains: psychiatric symptoms (disturbance of mental ability, depression, hallucinations, euphoria, reduced alertness, psychomotor slowing), cranial nerves, muscle tonus and strength, cerebellar function, pathological reflexes, sensibility, gait and stand, extrapyramidal symptoms, bradykinesia and functional performance. Neurological and neuropsychiatric manifestations were rated for severity on a three-point scale (1 = mild, 2 = moderate, 3 = severe). All items are weighted equally in the score A + B. The sum of individual neurological and neuropsychiatric symptoms and their severity was defined as the total score A + B. A higher score represents worse neurologic and neuropsychiatric outcome. This semiquantitative score was developed to provide a more objective evaluation of a subjectively influenced examination. To ensure consistency and reduce inter-examiner variability, all assessments were performed by a single, well-trained, and experienced investigator. Notably, this score has not yet undergone external validation.

Cognitive function was investigated using the Montreal Cognitive Assessment (MoCA) (12) and the health-related quality of life using the Short-Form Health Survey (SF-12) (13).

The Beck's Depression Inventory (BDI, scores >10 rated as abnormal) (14) was used for the assessment of depressive symptoms, and the Fatigue Impact Scale (FIS, scores >40 rated as abnormal) was used to assess fatigue (15).

We also assessed the general clinical outcome: the degree of disability was graded with the modified Barthel Index (score 0–100). Frailty status (robust, pre-frail or frail) was determined by the Frailty Index (FI), based on five domains (weight loss, exhaustion, grip strength weakness, gait speed, and physical activity level). Patients were classified as robust if they had none or only one impairment. Patients with two impairments were classified as pre-frail. Patients with three or more impairments were considered frail. In the structured interview we also obtained information on hospital readmissions, current medications, alcohol abuse, smoking habits, living conditions, mobility, stressful life events, education, symptoms of fatigue, depression, hallucinations, pain, headache, memory problems, sleeping disorders, personality changes, difficulty concentrating, and epileptic seizures at 1yFU.

### 2.3 Statistical analysis

Patient groups were classified according to the presence or absence of diagnosed POD. Group comparisons were performed using the Mann–Whitney *U*-test for continuous variables and the Pearson chi-squared test for group differences in categorical variables.

To assess whether POD is an independent risk factor for poor neuropsychiatric outcome and health-related quality of life (physical score), a multivariable linear regression analysis was conducted. The outcome parameters at 1yFU were used as dependent variates (MoCA at 1yFU, Score A + B and the Physical Component Summary of the SF-12). Parameters that differed significantly between patients with and without POD [age, difference between MoCA score at 1yFU and preoperative MoCA score (*Δ*MoCA)] and clinically relevant outcome factors (rehospitalization, NYHA classification) were included as independent variables. Due to a strong association with MoCA at 1yFU and Score A + B, *Δ*MoCA was excluded as an independent variable from the respective analyses.

Values of *p* < 0.05 were considered statistically significant. Statistical analyses were performed with SPSS for Windows (IBM Corp. Released 2021. IBM SPSS Statistics for Windows, Version 28.0. Armonk, NY: IBM Corp).

## 3 Results

### 3.1 Patient characteristics and prevalence of POD

A total of 179 patients received 1yFU after cardiac surgery or intervention. The median age of the cohort was 72.5 years. 35.8% of the patients were female (Table 1). 146 patients underwent elective cardiac surgery [cardiac valve surgery, coronary artery bypass graft (CABG), off-pump CABG, aortic surgery, combined CABG and cardiac valve surgery or transapical valve implantation], while 33 patients underwent transcatheter aortic valve replacement (TAVR) or catheter-based mitral valve reconstruction (MitraClip®, Abbott Vascular, Chicago, USA) (Table 2). Of these 179 patients, 43% (*n* = 77) had POD at the index event. During the year following the procedure, 61 patients required at least one hospital readmission (34.1%) and 19 patients required an ICU readmission (10.6%). Of the patients who were readmitted, 32.8% (*n* = 20) had more than one readmission to the hospital. The most common indications for rehospitalization were cardiac or other medical reasons (Table 3).

**Table 1:** Baseline characteristics according to the status of POD.

	Overall (*n* = 179)	POD (*n* = 77)	No POD (*n* = 102)	*p*
Demographics				
Age, yr, mean (±SD)	72.5 (11.6)	75.2 (9.5)	70.4 (12.6)	0.005*
Sex, male, *n* (%)	115 (64.2)	53 (68.8)	62 (60.8)	0.266
BMI, kg/m^2^, mean (±SD)	27.6 (5.2)	27.1 (4.2)	28 (5.8)	0.663
>12 years of education, *n* (%)	104 (58.1)	38 (49.4)	66 (64.7)	0.300
Comorbidities				
Diabetes mellitus, *n* (%)	56 (31.3)	27 (35.1)	29 (28.4)	0.343
Hypertension, *n* (%)	25 (14)	11 (13,3)	14 (13.7)	0.929
Coronary heart disease, *n* (%)	153 (85.5)	70 (90.9)	83 (81.4)	0.056
NYHA at 1yFU, *n* (%)				0.332
I	56 (31.3)	20 (26)	36 (35.3)	
II	99 (55.3)	44 (57.1)	55 (53.9)	
III	23 (12.8)	12 (15.6)	11 (10.8)	
Eversmoker, *n* (%)	100 (55.9)	42 (54,5)	58 (56.9)	0.953
Alcoholabuse, *n* (%)	19 (10.6)	7 (9.1)	12 (11.8)	0.429

The baseline characteristics contain demographic data and information on comorbidities. Statistical analysis was performed using Chi-Square test for categorical variables presented as percentage and Mann–Whitney-*U*-test for continuous variables presented as median with interquartile range (IQR). *P* < 0.05 was considered significant. BMI, body-mass-index; NYHA, New York Heart Association, POD, postoperative delirium.

*Indicates statistical significance (*p* < 0.05).

**Table 2:** Procedural characteristics and rehospitalization data.

	Overall (*n* = 179)	POD (*n* = 77)	No POD (*n* = 102)	*p*
Procedural characteristics				
Surgery type, *n* (%)				
TAVR/Mitral clip	33 (18.4)	13 (16.9)	20 (19.6)	0.121
Cardiac valve surgery	46 (25.7)	15 (19.5)	31 (30.4)	
CABG	55 (30.7)	21 (27.3)	34 (33.3)	
Off-pump CABG	13 (7.3)	9 (11.7)	4 (3.9)	
Aortic surgery	6 (3.4)	4 (5.2)	2 (2)	
Combined CABG and cardiac valve surgery	24 (13.4)	14 (18.2)	10 (9.8)	
Transapical valve implantation	2 (1.1)	1 (1.3)	1 (1)	
Length of operation, min, mean (±SD)	187.23 (81.43)	200.23 (86.48)	177.42 (76.36)	0.090
Total Erythrocyte concentrates, MD (IQR)	2 (0–4)	2 (1–5.5)	1 (0–3)	<0.001*
Rehospitalization data				
Readmitted patients, *n* (%)	61 (34.1)	29 (37.7)	32 (31.4)	0.379
Causes for rehospitalisation, *n* (%)				
Cardiologic/cardiothoracic surgery	27 (29)	10 (21.7)	17 (36.2)	
Surgical (other than cardiothoracic)	21 (22.6)	12 (26.1)	9 (19.1)	
Internal (other than cardiologic)	33 (35.5)	21 (45.7)	12 (25.5)	
Neurologic	4 (4.3)	1 (2.2)	3 (6.4)	
Other	8 (8.6)	2 (4.3)	6 (12.8)	

Procedural characteristics are details for cardiac surgery or percutaneous valve replacement. Rehospitalization data demonstrate readmissions to hospital between initial procedure and 1yFU. Statistical analysis was performed using Chi-Square test for categorical variables presented as percentage and Mann–Whitney-*U*-test for continuous variables presented as median with IQR. *P* < 0.05 was considered significant. TAVR, transcatheter aortic valve replacement; CABG, coronary artery bypass graft; POD, Postoperative delirium.

* Indicates statistical significance (*p* < 0.05).

**Table 3:** Neurologic and neuropsychiatric long-term outcome according to status of POD.

	Overall (*n* = 179)	POD (*n* = 77)	No POD (*n* = 102)	*p*
Score A + B, MD (IQR)	3 (1–7)	4.5 (2–9)	2 (0–6)	<0.001*
Score B, MD (IQR)	1 (0–2)	1 (0–2)	2 (1–3)	<0.001*
Score A, MD (IQR)	2 (0–6)	4 (1–6)	1 (0–5)	0.004*
Prevalence, *n* (%)				
Gait disturbance	71 (39.7)	38 (49.4)	33 (32.3)	0.148
Bradykinesia	61 (34.1)	36 (46.8)	25 (24.5)	0.008*
Dysdiadochokinesis	43 (24)	29 (37.7)	14 (13.8)	<0.001
Epicritic sensibility	58 (32.4)	27 (35.1)	31 (30.4)	0.466
Finger-nose	36 (20.1)	23 (29.9)	13 (12.8)	0.026*
Paresis	58 (32.4)	31 (40.3)	27 (26.5)	0.038*
MoCA preoperative, MD (IQR)	24 (22–26)	23 (21–25.5)	25 (23–26)	<0.001*
MoCA postoperative, MD (IQR)	25 (23–28)	24 (22–26.5)	26 (24–28)	<0.001*
Executive function/				
Visuospatial ability	4 (3–5)	4 (3–4)	4 (3–4)	0.001*
Animal naming	3 (3–3)	3 (3–3)	3 (3–3)	0.020*
Attention	6 (5–6)	6 (5–6)	6 (5–6)	0.112
Language abilities	2 (1–3)	2 (1–2)	2 (1–2)	0.001*
Abstraction	2 (2–2)	2 (2–2)	2 (2–2)	0.106
Short-term memory	3 (2–4)	2 (2–2)	2 (2–2)	0.003*
Orientation	6 (6–6)	6 (6–6)	6 (6–6)	<0.001*
*Δ*MoCA over 1 year, MD (IQR)	1 (0–3)	1 (−1–2)	2 (0–3)	0.027*
Depression, BDI >10, *n* (%)	36 (20.1)	15 (19.5)	21 (20.6)	0.855
Fatigue, FIS >40, *n* (%)	72 (40.2)	36 (46.8)	36 (35.3)	0.122
Barthel Index, mean (±SD)	95,3 (9.2)	93,4 (10.9)	96,7 (7.3)	0.025*
Frailty, *n* (%)				0.028*
Robust	73 (40.8)	23 (29.9)	50 (49)	
Pre-frail	72 (40.2)	35 (45.5)	37 (36.4)	
Frail	34 (19.1)	19 (24.7)	15 (14.7)	
SF-12, mean(±SD)				
Physical component	41.6 (10.5)	39.5 (10.4)	43.2 (10.4)	0.022*
Summary (PCS)				
Mental component	52.4 (8.5)	50.9 (9.2)	53.4 (7.9)	0.048*
Summary (MCS)				

Neuropsychiatric and neurological findings according to the status of POD are indicated. Statistical analysis was performed using Chi-Square test for categorical variables presented as percentage and Mann–Whitney-*U*-test for continuous variables presented as median with IQR. *P* < 0.05 was considered significant. SF-12, Short-Form-Health-Survey-12; BDI, Beck's Depression Inventory; FIS, fatigue impact scale; MoCA, Montreal Cognitive Assessment; POD, postoperative delirium. Score A covers neurological complaints, whereas score B covers neuropsychiatric symptoms. Score A + B includes both, neurological and neuropsychiatric symptoms.

* Indicates statistical significance (*p* < 0.05).

Patients were compared according to their POD status. Patients with POD were older (*p* = 0.005) and had worse preoperative cognitive performance [preoperative MoCA: POD 23 (IQR 21–25.5) vs. without POD 25 (IQR 23–26), *p* < 0.001]. Readmission rates were not significantly different between patients with or without POD (*p* = 0.379). Clinical characteristics grouped by POD status are shown in Table 1.

### 3.2 Long-term neurologic and neuropsychiatric symptoms

134 (74.9%) of 179 patients had at least one neurological or neuropsychiatric symptom one year after surgery or intervention. The most common neurological complaints were gait disturbance (*n* = 71), abnormal epicritic sensibility (*n* = 58), paresis (*n* = 58) and headache (*n* = 25). 41.9% of patients (*n* = 75) had chronic pain and 34.1% had sleep disturbances (*n* = 61) at 1yFU. Of these, 31 patients had difficulty falling asleep, 18 had difficulty staying asleep, and 12 had both problems. Patients experiencing chronic pain had a median score of 3 on the Numeric Pain Rating Scale (NPRS, 0–10). Comparing the median NPRS scores of patients with chronic pain with POD (*n* = 33, median score: 5) and without POD (*n* = 42, median score: 3), there was no significant difference (*p* = 0.088).

We compared the composite neurological and neuropsychiatric scores (score A + B) at 1yFU in patients with and without POD. In univariate analysis, patients with POD had higher score A + B, indicating worse neurologic and neuropsychiatric outcome (*p* < 0.001). Differences in neurological score (score A) and neuropsychiatric score (score B) remained for each category, with worse results in patients with POD (Table 3).

Multivariable linear regression analysis including the independent variables POD status, age, rehospitalization and NYHA classification revealed that POD, age, NYHA classification and rehospitalization were associated with a higher score A + B at 1 year, indicating a worse neurological and neuropsychiatric outcome (POD status: b:1.172; 95%-CI, 0.070–2.273, *p* = 0.037; age: b:0.134; 95%-CI, 0.086–0.182, *p* < 0.001; NYHA classification at 1 year: b:1.998; 95%-CI,1.169–2.828, *p* < 0.001; rehospitalization b:1.786; 95%-CI, 0.640–2.932, *p* = 0.002).

### 3.3 Cognitive function

The median preoperative MoCA score was 24 (IQR 22–26), while the median MoCA score at 1yFU was 25 (IQR 23–28, *p* < 0.001). When comparing 1yFU levels with baseline values the median MoCA score improved in both, patients with and without POD although the improvement was less apparent in the delirium group (*p* = 0.027). Among the individual MoCA items, executive function/visuospatial ability (*p* = 0.001), verbal ability (*p* = 0.001), short-term memory (*p* = 0.003) and orientation (*p* < 0.001) were the items with lower scores in patients with POD (Table 3). When POD status, age, rehospitalization and NYHA classification at 1yFU were included in a multivariable regression analysis, POD and NYHA classification were independently associated with worse MoCA scores at 1 year [POD status: b:-1.955, 95%-CI: −2.839-(−1.071), *p* < 0.001, age: b: −0.047, 95%-CI: −0.086-(−0.008), *p* = 0.018, NYHA classification: b: −1.059, 95%-CI: −1.727-(−0.391), *p* = 0.002, rehospitalization b: −0.625, 95%-CI: −1.545–0.295, *p* = 0.181].

### 3.4 Depressive symptoms and symptoms of fatigue

At 1yFU, 36 patients (20.1%) had abnormal BDI scores >10, indicating depressive symptoms. 72 patients (40.2%) had abnormal FIS scores >40, indicating the presence of fatigue. When compared bilaterally, the prevalence of abnormal BDI and FIS scores was not significantly different between patients with and without POD (Table 3).

### 3.5 Functional outcomes and HRQoL

Patients with POD had more frailty (*p* = 0.028) and a lower Barthel Index (*p* = 0.025) (Table 3). Both the SF-12 Physical Component Summary (*p* = 0.022) and SF-12 Mental Component Summary (*p* = 0.048) were significantly lower in patients with POD. Multivariable regression analysis demonstrated an association of POD, NYHA classification at 1yFU and rehospitalization with the SF-12 physical component summary scores one year after cardiac surgery or intervention [POD status: b:−2.847, 95%-CI: −5,670-(−0,024), *p* = 0.048; NYHA classification: b:−6.176, 95%-CI: −8.309-(−4.043), *p* < 0.001; rehospitalisation b:−3.826, 95%-CI: −6.778-(0.875), *p* = 0.011; age: b:−0.038, 95%CI: −0.166–0.090, *p* = 0.56; ΔMoCA: b:−0.061, 95%CI: −0.642–0.52, *p* = 0.836).

### 3.6 Neurologic and neuropsychiatric outcome in subgroups of POD

A subgroup analysis of delirium subtypes (hyperactive, hypoactive and mixed subtype) was performed (Supplementary Table S1). No significant differences were observed between these subtypes regarding neurologic and neuropsychiatric outcomes.

## 4 Discussion

The aim of the present study is to illustrate the neurological and neuropsychiatric outcome one year after cardiac surgery or percutaneous valve replacement. In this monocentric, prospective, observational study, we were able to show, that neurological and neuropsychiatric disorders are common in patients with cardiac pathologies and a history of cardiac surgery or intervention, and that POD is independently associated with the occurrence of these impairments. Patients who developed POD also had worse cognitive function compared with those who did not, as shown by MoCA scores before and one year after surgery. Health-related quality of life was worse in patients with a history of POD during their cardiac procedures, whereas symptoms of depression, fatigue or rehospitalization rates did not show any association. Notably, no significant differences in outcomes were observed between subgroups of POD (hypoactive, hyperactive, and mixed).

### 4.1 Association of POD with neurologic and neuropsychiatric long-term outcome

Consistent with our findings, in a recent retrospective cohort study, including 4,033 patients, Brown et al. discovered an association between POD and new-onset neurocognitive disorders (such as dementia or memory impairment) one year after their ICU stay (16). However, no association was found between POD and depressive episodes, anxiety, and trauma-and-stressor-related disorders. In contrast to our study, the information on the patients’ neuropsychiatric disorders was obtained retrospectively from the medical records, which does not allow any conclusions to be drawn about the patients’ clinical characteristics or even individual symptoms.

In the present study, we found that both, patients with and without POD, complained of various neurological symptoms such as gait disturbance, bradykinesia and paresis one year after surgery. Many patients also experienced symptoms of depression (20.1%) and fatigue (40.2%) at 1yFU. These findings underscore the need for outpatient follow-up for these patients after discharge from the hospital. Various concepts of post-ICU follow-up services are currently being investigated to address various long-term complaints of these patients (17, 18). They vary in frequency of consultation (e.g., monthly, once after six months), specific interventions, and patient population (all patients after ICU stay vs. at-risk patients), but they only partially address neuropsychiatric complaints such as depression or cognitive function (17). POD is one of several factors influencing long-term neurologic and neuropsychiatric outcomes. These outcomes are determined by a complex interplay of multiple elements, including postoperative neurologic events, overall clinical course, and rehospitalizations. Moreover, the majority of patients participated in cardiac rehabilitation, which may be a significant contributor and could have exerted a beneficial effect on neurologic outcomes. Notwithstanding these considerations, our findings indicate that POD serve as a predictor for long-term outcome parameters and could facilitate the identification of patients who may benefit from structured follow-up and intensified postoperative care.

In addition, no significant differences regarding neurologic and neuropsychiatric outcomes are observed between the subtypes of POD (hyperactive, hypoactive and mixed subtype). However, due to smaller subgroup sizes, these results are not yet conclusive. Larger sample sizes will be required for a more robust evaluation of these subgroups.

### 4.2 Potential mechanisms linking POD with neurologic and neuropsychiatric symptoms

There are several interpretations and explanations for the association of POD with long-term neurological and neuropsychiatric outcomes. First, POD may reflect pre-existing impaired cerebral function or cognitive reserve prior to surgery or intervention. This pre-existing impairment may be revealed by the effects of non-specific insults, such as surgery-induced neuroinflammation and anesthesia, on the vulnerable brain (19). Affected patients are more likely to suffer from POD and might carry an increased risk of developing neurological and neuropsychiatric symptoms in the long term. The close association of cognitive decline with age and the higher degree of heart failure may reinforce these observations (20, 21).

A second hypothesis is that cerebral microembolization and hypoperfusion during cardiac surgery might significantly damage the brain and thus contribute to both, POD and worse long-term neuropsychiatric outcome (22). Of note, the impact of postoperative hyperperfusion has also been described as a detrimental condition with delayed cognitive recovery, respectively (23). In addition, research indicates that after surgical trauma, peripheral inflammatory factors cross the blood-brain barrier and consecutively trigger post-surgical neuroinflammation, which can cause synaptic dysfunction and neuronal death, ultimately leading to both POD and worse cerebral performance (24, 25).

A third approach is that psychiatric stressors are a strong trigger for prolonged neurological and neuropsychiatric symptoms. In 559 patients after elective noncardiac surgery, Drews et al. investigated the association between the occurrence of POD and posttraumatic stress disorder (PTSD) 3 months after surgery using the PTSS-14 questionnaire. They demonstrated that POD was a risk factor for PTSD at 3 months after surgery (26). A systematic review illustrated the relationship between POD and symptoms of depression (7). The authors showed that there are controversial data on the relationship of POD and symptoms of depression and concluded that they might be risk factors for each other. In our study, we found no significant differences in the occurrence of symptoms of depression as assessed by the BDI in relation to POD status. However, previous studies have shown higher rates of psychiatric comorbidities such as depression and anxiety in patients with cardiac disease (27, 28). This is consistent with the high prevalence of depression symptoms in our patient cohort (BDI >10, *n* = 36, 20.1%).

Regarding the high prevalence of depression in patients with cardiac diseases, one of the proposed mechanisms focuses on dysregulation of the hypothalamic-pituitary-adrenal axis. Emotional stress is associated with increased cortisol levels and plays an important role in the pathophysiology of depression (29, 30). Elevated cortisol levels can lead to decreased sensitivity of glucocorticoid receptors in the hypothalamic-pituitary-adrenal axis. Therefore, a compromised negative feedback loop may result in cortisol hypersecretion (31). These high cortisol levels are associated with cardiovascular risk factors, such as hypertension and dyslipidemia, and thus contribute to cardiovascular disease (32, 33).

Behavioral factors may also play a role. In a prospective study of 1,017 outpatients with stable coronary artery disease, the authors found an association between physical inactivity in patients with symptoms of depression and adverse cardiac events in their long-term follow-up after a mean of 4.8 years (34).

### 4.3 POD and quality of life and rehospitalization after cardiac surgery

An association between POD and a decreased quality of life, as measured by the Medical Outcomes Study Short-Form General Health Survey, has also been suggested (35). In the present analysis we found lower scores on the Physical Component Summary of the SF-12, indicating a worse quality of life one year after surgery or intervention in patients with POD compared to patients without POD. This observation has been reported in several studies investigating quality of life after cardiac surgery. In patients with POD, Koster et al. observed a reduced quality of life at 6 months after cardiac surgery using the SF-36 (4), Loponen et al. found lower health-related quality of life in patients with POD up to 3 years after cardiac surgery (36). To address these issues, Ma et al. suggested that comprehensive rehabilitation combined with an educational program could help to reduce the rates of depression and anxiety and might improve quality of life after surgery in patients who underwent CABG (37).

Regarding hospital readmission, there was no significant difference between patients with and without POD in our study. Because the reasons for the readmission of patients in our study were diverse (Table 3), a close pathophysiologic link between POD and readmission is questionable.

### 4.4 Postoperative cognitive dysfunction and POD

Regarding cognitive performance, particularly after cardiac surgery, postoperative cognitive dysfunction (POCD) is a common complication with a prevalence ranging from 10 to 54% within the first few weeks (38). The term is used to describe an impairment of cognitive functions, including for example the executive function or attention, following surgery (39, 40). In the present study we found that the median MoCA score was higher in all patients one year after elective cardiac surgery compared to baseline. However, when comparing the delirious and non-delirious groups, the recovery was less pronounced in the group with POD. Several studies have already suggested a recovery of cognitive performance over time compared to preoperative baseline cognitive levels. Newman et al. found a decrease in the rate of cognitive dysfunction in patients undergoing CABG from 3 months to 6 months after surgery, and then again a higher rate of cognitive dysfunction at 5 years after surgery (41). Sauër et al. also demonstrated recovery of cognitive function one year after cardiac surgery (42).

One explanation for the improved overall MoCA score with 1yFU in our study is that it may be an effect of the testing environment. Preoperatively patients were tested in the hospital three to one day prior to their intervention or surgery, whereas during their follow-up visit, they were tested at home or in the hospital in a calm environment. Hence, anxiety and stress prior to the upcoming surgery or intervention might have affected the preoperative test results. Contradicting this view, data from another group of patients with recent brain injury showed that MoCA scores were not dependent on the testing environment, either in the ICU or in the office (43).

Also, Sacynzky et al. assessed cognitive function in patients undergoing CABG or valve replacement using MMSE (3). They reported that fewer patients with POD had returned to baseline cognitive function at 6 months compared with patients without POD. At 12 months, the proportion of patients returning to pre-surgical cognitive function did not differ between the two groups.

Another explanatory approach might be that improved cardiac output leading to better cerebral perfusion after cardiac surgery or percutaneous valve replacement might improve cognitive performance in the 1yFU (44). However, cardiac parameters were not assessed at the 1yFU in our cohrt.

In the cognitive domain, advanced age and depression must also be considered as independent risk factors for cognitive decline, as these factors may additionally influence the long-term cognitive outcome (45, 46).

### 4.5 Diagnostics, treatment and aftercare of POD

Given the high prevalence of POD after cardiac surgery and its short and long-term adverse outcomes, preventive and therapeutic strategies should be considered. Establishing routine screening for delirium in postoperative care has been recommended in various guidelines, but adherence to this recommendation is still insufficient (47).

Follow-up care (e.g., mental health assessments) might be useful to identify patients experiencing neuropsychiatric symptoms, such as cognitive impairment, depression or fatigue after cardiac surgery or percutaneous valve replacement. As part of an enhanced after-care process, the provision of psychological support may be helpful.

## 5 Limitations

There are several limitations that deserve to be mentioned. Due to its observational design, this study cannot generate proof of causality for the association of POD with long-term neurologic and neuropsychiatric outcomes. A notable limitation of our study is the absence of preoperative psychiatric and neurologic assessments, which precludes differentiation between pre-existing neurological and neuropsychiatric symptoms and new postoperative complaints. Patients exhibiting lower baseline cognitive or psychiatric function may be at an increased risk for developing POD or experiencing unfavorable long term outcomes. Future research should aim to collect standardized preoperative neurologic and neuropsychiatric baseline data in order to precisely differentiate the impact of delirium from that of preexisting impairments. Another limitation is that we did not include a non-surgical comparison group in our study to compare the prevalence of complaints in both groups. Also, this study was conducted at a single center and involved a relatively small sample size (*n* = 179), of which 43 percent developed postoperative delirium (POD). Consequently, the generalizability of these results may be limited.

However, strengths of this study include the prospective design and the preoperative cognitive assessment in order to compare pre- and postoperative results. As mentioned above, a number of patients were lost to follow-up. These patients may have been in a worse condition and less likely to participate in the 1-year follow-up.

## 6 Conclusion

Long-term neurological and neuropsychiatric impairments are common after cardiac surgery and percutaneous valve replacement with 1yFU. These symptoms, as well as HRQoL and cognitive function, are independently associated with the occurrence of POD. The presence of POD may be a relevant prognostic marker to identify patients who may benefit from specific follow-up services.

## Data Availability

The raw data supporting the conclusions of this article will be made available by the authors upon reasonable request.
