# Supplementary material for: Impact of postoperative delirium on long-term neurologic and neuropsychiatric outcome after cardiac surgery or percutaneous valve replacement–a prospective observational study
Source: Front Cardiovasc Med. 2025 Nov 24;12:1635225. doi: 10.3389/fcvm.2025.1635225 (PMC12682873; doi:10.3389/fcvm.2025.1635225)
Supplement: Supplementary file 1 [file Table1.pdf]

Supplementary Table 1. Neurologic and neuropsychiatric long-term outcome according to subtype of POD

	**Hyperactive subtype (n=42)**	**Hypoactive subtype (n=13)**	**mixed subtype (n=22)**	**p**
**Demographics**				
**Age, yr, mean (±SD)**	75.86 (10)	69.85 (10.7)	77.18 (6.5)	0.104
**Sex, male, n (%)**	31 (73.8)	8 (61.5)	14 (63.6)	0.368
**BMI, kg/m^2, mean (±SD)**	27.1 (4.6)	28.1 (3.7)	26.7 (3.9)	0.772
**Rehospitalizations and Neurologic and neuropsychiatric long-term outcome**				
**Readmitted patients, n (%)**	15 (34.9)	5 (38.5)	9 (40.1)	0.918
**Score A+B, MD (IQR)**	4.5 (2-8.25)	2 (1.5-11)	6.5 (3.75-9)	0.323
**Score B, MD (IQR)**	1 (0.75-3)	2 (0.5-2.5)	2 (1-2.25)	0.730
**Score A, MD (IQR)**	3 (1-6)	1 (0-8.5)	4 (2.5-7)	0.188
**MoCA preoperative, MD (IQR)**	23 (20.75-26)	23 (22.5-25.5)	23.5 (21-25)	0.784
**MoCA postoperative, MD (IQR)**	23.5 (21-27.25)	24 (20.5-26)	24 (23-27)	0.735
**ΔMoCA over 1 year, MD (IQR)**	1 (-1-2)	1 (-2.5-1.5)	1 (-0.25-4)	0.420
**Depression, BDI >10, n (%)**	7 (16.7)	5 (38.5)	3 (13.6)	0.159
**Fatigue, FIS >40, n (%)**	18 (42.9)	8 (61.5)	10 (45.5)	0.493
**SF-12, mean(±SD)**				
**physical component summary (PCS)**	41.5 (10.7)	37.5 (12.1)	36.9 (8.1)	0.222
**mental component summary (MCS)**	51.2 (9.4)	48.8 (9.7)	51.8 (8.6)	0.589

Supplementary Table 1: Demographics and long-term outcome data are summarized according to subtype of POD. Statistical analysis was performed using Chi-Square test for categorical variables presented as percentage and Kruskal-Wallis-test for continuous variables presented as median with IQR. P<0.05 was considered significant. BMI, Body-Mass-Index; POD, postoperative delirium. SF-12, Short-Form-Health-Survey-12; BDI, Beck's Depression Inventory; FIS, Fatigue Impact Scale; MoCA, Montreal Cognitive Assessment; POD, Postoperative delirium. Score A covers neurological complaints, whereas score B covers neuropsychiatric symptoms. Score A+B includes both, neurological and neuropsychiatric symptoms.
