# Supplementary material for: Impact of postoperative delirium on long-term neurologic and neuropsychiatric outcome after cardiac surgery or percutaneous valve replacement–a prospective observational study
Source: Front Cardiovasc Med. 2025 Nov 24;12:1635225. doi: 10.3389/fcvm.2025.1635225 (PMC12682873; doi:10.3389/fcvm.2025.1635225)
Supplement: Supplementary file 2 [file Table2.pdf]

Supplementary Table 2. Examination protocol for neurologic and neuropsychiatric outcome (Score A+B)

**Score A**	
Cranial Nerves	Yes / No
Muscle and strength	
Hypotonia	0-3
Spasticity	0-3
Paresis	0-3
Impaired position maintenance	0-3
Cerebellar function	
Positive rebound phenomenon	0-3
Dysdiadochokinesis	0-3
Finger-Nose-test	0-3
Heel-Knee-test	0-3
Pathological reflexes	
Hyperreflexia	0-3
Hyporeflexia	0-3
Pyramidal signs	0-3
Sensibility	0-3
Gait and stand	0-3
Spastic gait	0-3
Ataxic gait	0-3
Romberg test	0-3
Extrapyramidal symptoms	
Tremor	0-3
Rigidity	0-3
Bradykinesia	
Bradykinesia	0-3
Gait disturbance	0-3
Posture	0-3
Arising from chair	0-3
Postural stability	0-3
Functional performance	
Alternative movements	0-3
Facial expression	0-3
Sialorrhea	0-3
Speech disorder	0-3
Dyskinesia	0-3
Dystonic postures	0-3
**Score B**	
Disturbance of mental ability	0-3
Hallucinations	Yes / No
Depression	0-3
Euphoria	0-3
Reduced alertness	0-3
Psychomotor slowing	0-3

Supplementary Table 2: Examination protocol used for the evaluation of neurological complaints (Score A) and neuropsychiatric symptoms (Score B) at 1yFU. Neurological and neuropsychiatric manifestations were rated for severity on a three-point scale (1 = mild, 2 = moderate, 3 = severe). A "yes" counts as one point in the scoring system. A higher score indicates a worse neurological and neuropsychiatric outcome.
